# Cell-Free DNA as a Prognostic Biomarker in Oral Carcinogenesis and Oral Squamous Cell Carcinoma: A Translational Perspective

**DOI:** 10.3390/cancers17142366

**Published:** 2025-07-16

**Authors:** Pietro Rigotti, Alessandro Polizzi, Vincenzo Quinzi, Andrea Blasi, Teresa Lombardi, Eleonora Lo Muzio, Gaetano Isola

**Affiliations:** 1 Department of General Surgery and Surgical-Medical Specialties, School of Dentistry, University of Catania, 95124 Catania, Italy; pietro.rigotti1999@libero.it (P.R.); gaetano.isola@unict.it (G.I.); 2 Department of Life, Health & Environmental Sciences, Postgraduate School of Orthodontics, University of L’Aquila, 67100 L’Aquila, Italy; vincenzo.quinzi@univaq.it; 3 Department of Neurosciences, Reproductive and Odontostomatological Sciences, University of Naples Federico II, 80138 Naples, Italy; andrea.blasi@unina.it; 4 Department of Health Sciences, Magna Græcia University, 88100 Catanzaro, Italy; drteresalombardi@libero.it; 5 Department of Clinical and Experimental Medicine, University of Foggia, Via Rovelli 50, 71122 Foggia, Italy; eleonoralomuzio@gmail.com; 6 International Research Center on Periodontal and Systemic Health “PerioHealth”, University of Catania, 95124 Catania, Italy

**Keywords:** oral squamous cell carcinoma, oral potentially malignant disorders, cell-free DNA, liquid biopsy, oral carcinogenesis

## Abstract

Oral squamous cell carcinoma (OSCC) remains one of the most common malignancies in the head and neck region, often preceded by a spectrum of oral potentially malignant disorders (OPMDs). Despite advances in diagnostic methods, reliable and non-invasive biomarkers for early detection and prognostic stratification are still lacking. In recent years, circulating cell-free DNA (cfDNA) has emerged as a promising liquid biopsy tool in several solid tumors, offering insights into tumor burden, heterogeneity, and molecular dynamics. However, its application in oral oncology remains underexplored. This study aims to review and discuss the current evidence on cfDNA quantification and mutation analysis (including TP53, NOTCH1, and EGFR) in patients with OPMDs and OSCC. Particular attention is given to cfDNA fragmentation patterns, methylation signatures, and tumor-specific mutations as prognostic and predictive biomarkers. Moreover, we highlight the challenges in standardizing pre-analytical and analytical workflows in oral cancer patients and explore the potential role of cfDNA in monitoring oral carcinogenesis. Understanding cfDNA dynamics in the oral cavity might offer a novel, minimally invasive strategy to improve early diagnosis, risk assessment, and treatment decision-making in oral oncology.

## Simple Summary

Oral cancer is a serious and often aggressive disease that can develop from earlier changes in the mouth, known as oral potentially malignant disorders (OPMDs). Detecting which OPMDs will progress to cancer remains a major clinical challenge. In recent years, scientists have discovered that small fragments of DNA released by cancer cells—called cell-free DNA (cfDNA)—can be found in the blood or saliva of patients with solid tumors. This type of “liquid biopsy” offers a non-invasive way to monitor cancer development and response to treatment. In this article, we explore how cfDNA could be used as a biomarker to detect early oral cancer, predict disease progression, and guide personalized treatment strategies. Understanding how cfDNA changes during oral carcinogenesis could help clinicians better identify high-risk patients and improve survival outcomes through earlier, targeted interventions.

## 1. Introduction

Oral squamous cell carcinoma (OSCC) is the most common malignant neoplasm of the oral cavity [1], accounting for over 90% of all oral cancers [2,3,4,5]. Globally, oral cancer represents a significant public health issue and it is ranked among the most prevalent cancers worldwide [6,7], affecting approximately 600,000 individuals annually [8]. According to recent data, including GLOBOCAN 2020, the global incidence of lip and oral cavity cancer is estimated at around 377,000 new cases per year [9], while broader estimates from 2021 report approximately 890,000 newly diagnosed head and neck cancers and 450,000 cancer-related deaths each year [10]. Specifically for oral cancer, GLOBOCAN 2018 recorded approximately 500,550 cases and 177,384 deaths worldwide [6], with 178,000 OSCC-related deaths reported in 2020 [11]. OSCC can affect various anatomical subsites, including the gingiva, tongue, lips, and floor of the mouth [7], and is significantly more common in individuals over the age of 45, with a higher incidence observed in women [12].

Despite advances in molecular alterations [13], diagnostic techniques, and therapeutic strategies, the five-year survival rate for OSCC remains relatively low, estimated at around 50–60% [8] or approximately 62% [3], with only marginal improvements over the past years, increasing by less than 1% annually between 2005 and 2017 [5]. This high morbidity and mortality are primarily attributed to late-stage diagnosis, locoregional recurrence, and treatment resistance [8]. In contrast, early-stage detection of OSCC significantly improves prognosis, with stage I patients showing survival rates over 85% [5], compared to approximately 20% for those diagnosed at stage III or IV [7]. These figures underscore the critical importance of early diagnosis in improving clinical outcomes for OSCC patients [8,14].

The development of OSCC may be preceded by a group of clinical conditions known as oral potentially malignant disorders (OPMDs), which carry an increased risk of progression to cancer [2,15]. Common examples of OPMDs include oral leukoplakia (OLK), oral lichen planus (OLP), erythroplakia, and oral submucous fibrosis (OSF). The overall malignant transformation rate of OPMDs is estimated to be around 7.9% [9]. Moreover, although oral dysplasia is graded histologically, histopathological assessment alone does not reliably predict the clinical behavior or malignant potential of these lesions [5].

While tissue biopsy provides the definitive diagnosis for OSCC and OPMDs [6], these conditions are often diagnosed at advanced stages due to asymptomatic early disease [16]. Therefore, the identification of reliable biomarkers is essential for early diagnosis, prognosis, and treatment response prediction [8]. Biomarkers derived from various biological fields, such as genomics, proteomics, and metabolomics, play a critical role and may help to forecast the probability of OSCC development and/or progression [5]. The discovery of dependable biomarkers obtainable through non-invasive methods, for instance, from saliva, could significantly enhance OSCC management [3,5]. Although many potential biomarkers, both non-invasive (saliva and blood) and invasive (biopsy), have been identified over the years, few have been incorporated into standard clinical practice [7]. The discovery of novel biomarkers holds the promise to improve early OSCC diagnosis and patients’ prognosis [17]. For this reason, there is an urgent need for cost-effective diagnostic methods with high sensitivity and specificity and for more accurate biomarkers capable of predicting the progression of oral dysplasia to cancer, ultimately enabling better clinical management of these lesions [5,6].

In this context, liquid biopsy is emerging as a novel tool [18], offering promising prospects for the early diagnosis of oral cancer [19,20]. Liquid biopsy represents a minimally invasive and repeatable approach that enables the analysis of biomarkers released into body fluids, such as blood and saliva. Among the various components analyzed through liquid biopsy, circulating cell-free DNA (ccfDNA) has garnered particular interest due to its potential clinical relevance [18].

cfDNA consists of extracellular DNA fragments that freely circulate in the bloodstream and other body fluids [21]. While a significant portion of cfDNA is released by apoptotic cells, a crucial fraction, known as circulating tumor DNA (ctDNA), originates from malignant cells and carries tumor-specific genetic and epigenetic alterations, including somatic mutations and DNA methylation patterns [22]. The analysis of cfDNA, particularly ctDNA, through liquid biopsy offers several advantages: early tumor detection, assessment of tumor heterogeneity, prognostic evaluation, monitoring of treatment response, and real-time detection of recurrence [19].

**Figure 1:**
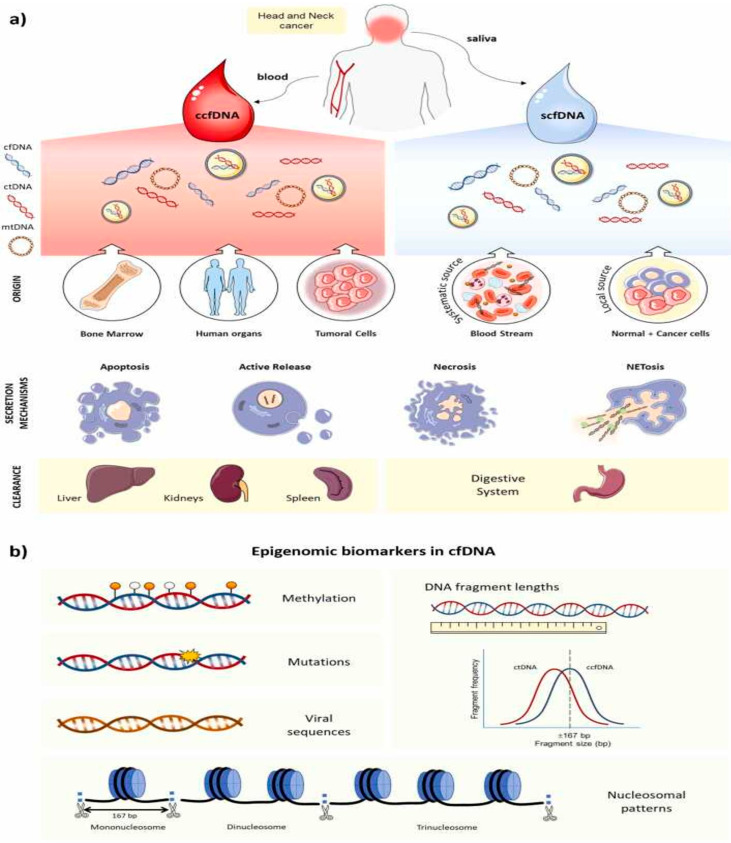
Overview of cell-free DNA (cfDNA) in head and neck cancer. (**a**) Origin, release, and clearance: cfDNA derives from normal and tumor cells via apoptosis, necrosis, active secretion, or NETosis. Circulating cfDNA (including ctDNA) originates largely from bone marrow, various organs, and tumor sites and is cleared by the liver, kidneys, and spleen. In saliva, cfDNA arises both locally—from shed oral epithelial cells—and systemically—via salivary-gland transudation—and is ultimately excreted through the digestive tract. (**b**) Epigenomic biomarkers: cfDNA carries tumor-specific alterations (mutations, methylation changes, and viral sequences) and bears fragmentomic and nucleosome patterns, providing insights into gene regulation and disease processes. From [24] with permission.

In oral cancer, liquid biopsy using samples, such as peripheral blood and saliva, may significantly enhance current diagnostic strategies by enabling earlier detection and dynamic disease monitoring [18]. Despite its huge potential, the full integration of cfDNA-based liquid biopsy into clinical practice in oral oncology still requires further investigation to address challenges related to sensitivity, specificity, and methodological standardization (Figure 1) [23].

In this regard, this review aims to provide a translational perspective on the role of cfDNA as a prognostic biomarker in oral carcinogenesis and OSCC, highlighting current evidence and future directions in this evolving field.

## 2. Overview of Cell-Free DNA

Cell-free DNA (cfDNA) refers to extracellular DNA molecules circulating within various bodily fluids [25]. These molecules represent a highly dense and multidimensional information repository, acting as a “biological mirror” reflecting the state and dynamics of genomes within the body [26]. The analysis of cfDNA has emerged as a non-invasive or minimally invasive approach, commonly referred to as “liquid biopsy,” offering significant potential in various clinical fields, including oncology [25,27].

### 2.1. Origin, Biological Features, and Mechanism Release in Cancer of cfDNA

The cfDNA molecules found in circulation are actively and/or passively released from cells [25]. In healthy individuals, the primary source of cfDNA is the hematopoietic system. However, in various physiological and pathological conditions, such as cancer, affected tissues significantly contribute to the cfDNA pool [28]. This fraction of cfDNA originating from tumors is specifically known as circulating tumor DNA (ctDNA) [29].

CfDNA fragments are generally short, non-randomly fragmented molecules [30], and the release of cfDNA into circulation is believed to occur mainly through apoptosis, necrosis, and active secretion [31]. A prevalent size of cfDNA is approximately 166–175 base pairs (bp), reflecting the length of DNA wrapped around nucleosomes [32]. Fragments released from apoptotic normal cells and blood cells are typically at around 160–180 bp in length, reflecting nucleosomal packaging [29,33]. However, cfDNA fragment length can vary, ranging from 40 bp up to more than 10 kilobases (kb), with longer fragments often derived from necrotic cells [32]. The pattern of apoptotic fragmentation is usually reflected in the size of cfDNA, yielding fragments of approximately 167 bp, corresponding to nucleosomal DNA plus a linker segment [29]. The ratio of longer to shorter cfDNA fragments has been proposed as a measure of cfDNA integrity (cfDI) [33]. Beyond passive release via cell death, active cellular secretion is another proposed mechanism for cfDNA generation [32]. Research, particularly in breast cancer cell lines, indicates that cells can actively release cfDNA, not exclusively through exosomes [34]. The exact mechanisms underlying cfDNA production and release are still being studied [32].

Importantly, cfDNA in circulation carries genetic information from its originating cells, including mutations, copy number alterations, chromosomal rearrangements, and epigenetic status like hyper/hypo-methylation. Carrying both genetic and epigenetic information, cfDNA represents a valuable source for molecular profiling [25].

### 2.2. cfDNA Detection in Blood vs. Saliva: Advantages and Disadvantages

CfDNA is present in various body fluids, including blood, saliva, urine, cerebrospinal fluid (CSF), and others [25,35]. For cancer diagnostics, blood plasma or serum has been the most widely studied source of cfDNA [27]. It is generally considered convenient for sampling, and cfDNA in plasma is relatively stable and straightforward to extract [36].

However, saliva is emerging as a promising alternative for liquid biopsy, particularly relevant for oral carcinogenesis and head and neck cancers. The collection of saliva is convenient, non-invasive, and painless, allowing for repetitive sampling [29,37]. Saliva contains biomarkers that diffuse from the circulation and are also shed directly from tumors or affected tissues in the oral cavity [38]; thus, thanks to this dual origin, saliva has the potential for the early detection of oral diseases [29].

While saliva offers ease of collection, a study has shown that salivary cfDNA concentration can be significantly lower than plasma cfDNA concentration. For some cancers, like non-small cell lung cancer, the concentration of salivary cfDNA may not be suitable for quantitative diagnostic analysis. However, salivary cfDNA can still be valuable for qualitative analysis, such as detecting specific mutations like EGFR mutations [39]. The yield of ctDNA can impact assay sensitivity, highlighting the importance of optimizing pre-analytical and analytical procedures for both blood and saliva samples [29].

However, this lower yield of salivary cfDNA can reduce analytical sensitivity, particularly when screening for early-stage or low-burden lesions. Studies have demonstrated that standard quantitative assays may fail to detect tumor-derived cfDNA in saliva when total cfDNA levels fall below assay limits of detection, potentially leading to false negatives in early disease. To overcome this, highly sensitive methods—such as digital PCR or targeted ultra-deep sequencing—are often required for salivary cfDNA analysis in early-stage OSCC and OPMDs, and assay performance should be validated with spiked-in controls to determine the minimal detectable tumor fraction [40].

Despite the potential challenges, salivary biomarkers, including cfDNA and circulating tumor DNA, show promise for the early detection of head and neck cancers, although large prospective clinical trials are still needed [29], and cfDNA analyses are increasingly seen as valuable tools for monitoring patients and offering personalized care [27].

## 3. CfDNA in Oral Squamous Cell Carcinoma

### 3.1. cfDNA Levels and Tumor Burden Correlation

Two studies of Lin et al. have investigated the presence and concentration of cfDNA in the plasma and saliva of OSCC patients as a potential indicator of tumor burden and disease progression. In patients with OSCC, plasma cfDNA concentrations have been reported to be significantly elevated compared to healthy controls [41,42]. For instance, one of these two studies observed a mean cfDNA concentration of 53.1 ± 6.69 ng/mL in OSCC patients compared to 24.0 ± 3.33 ng/mL in controls. This elevated plasma cfDNA level has shown correlation with larger tumor size (TNM T stage), cervical lymph node metastasis (N status), and advanced OSCC stage. While total cfDNA levels may not always be significantly elevated in early lesions or after surgery, cfDNA has been reported as a highly sensitive genetic biomarker reflecting tumor burden and genetic dynamics in various cancers, including OSCC. The accessible nature of plasma cfDNA makes it a potentially valuable tool for both diagnosis and prognosis in OSCC [42].

### 3.2. Gene Mutations of cfDNA

**Figure 2:**
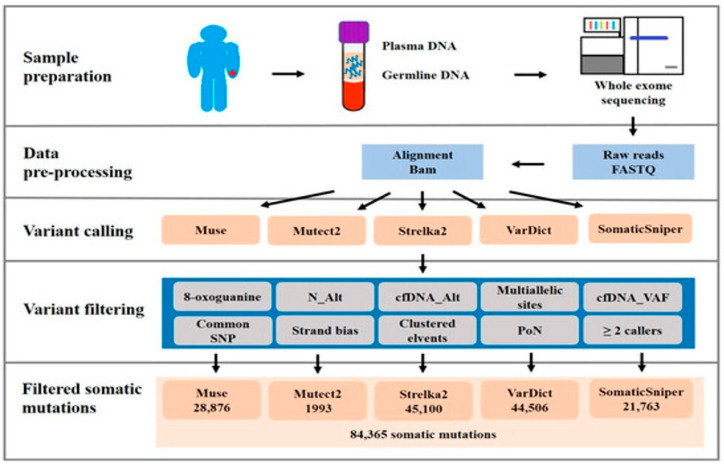
Flowchart illustrating the whole-exome sequencing (WES) analysis of cfDNA. From [41], reused under CC-BY 4.0.

Analyzing gene mutations in cfDNA provides a non-invasive way to profile the genomic alterations present in a tumor [41]. TP53 mutation is recognized as a driver mutation in oral carcinogenesis and is one of the most frequently explored gene mutations in ctDNA from Head and Neck Squamous Cell Carcinoma (HNSCC) patients [43,44]. While OSCC can have relatively low cfDNA shedding and mutations at low allele frequencies, detection of TP53 target hotspot somatic mutations in cfDNA from OSCC patients has been shown to be technically feasible, for instance, using techniques like droplet digital polymerase chain reaction (ddPCR). Moreover target somatic mutations detected in cancerous DNA and cfDNA can be related to cervical lymph node metastasis [44]. Beyond TP53, comprehensive genomic profiling of plasma cfDNA in patients with metastatic OSCC has identified other frequently mutated genes, including TTN, PLEC, SYNE1, and USH2A. Known driver genes such as KMT2D, LRP1B, TRRAP, and FLNA were also found to be significantly and frequently mutated. Additionally, novel mutated genes like CCDC168, HMCN2, STARD9, and CRAMP1 were significantly present in metastatic OSCC patients. Genes like RORC, SLC49A3, and NUMBL were most frequently mutated in patients with distant metastatic OSCC. Whole-exome sequencing (WES) of plasma cfDNA and paired whole blood samples allows for the identification of somatic mutations and analysis of mutation burden, which has been significantly associated with clinical staging and distant metastasis status in OSCC (Figure 2) [41]. Analysis of public databases and specific OSCC cohorts revealed frequently mutated genes like TP53, FAT1, CASP8, CDKN2A, NOTCH1, PIK3CA, and HRAS [45]. This molecular profiling of cfDNA provides insights into the genomic landscape of OSCC and may help in designing targeted therapies and patient stratification [41].

### 3.3. CfDNA Fragmentation and Integrity as Prognostic Markers

The process by which cfDNA is released, particularly from malignant cells undergoing necrosis, often results in incompletely digested DNA fragments that are longer than those typically produced by apoptosis in healthy individuals. This difference in fragment size distribution can be assessed by measuring cfDNA integrity, commonly calculated as the ratio of longer DNA fragments to shorter fragments. Specific short interspersed elements, such as ALU115 and ALU247 fragments, are frequently used to assess cfDNA integrity [46,47]. A higher cfDNA integrity index is often associated with malignancy; indeed, in oropharyngeal squamous cell carcinoma (OPSCC), a subtype of HNSCC, significantly higher cfDNA integrity has been observed compared to normal controls. This higher integrity in OPSCC was found to be associated with nodal status, and the combination of ALU115 quantity with cfDNA integrity enhanced diagnostic sensitivity [48]. Similarly, in laryngeal squamous cell carcinoma (LSCC), significantly higher levels of plasma cfDNA (ALU115 and ALU247) and cfDNA integrity index were detected in patients compared to non-tumor individuals. These findings suggest that cfDNA integrity can differentiate cancer patients from healthy controls [47]. While studies directly linking cfDNA integrity in saliva to OSCC prognosis are preliminary, salivary cfDNA integrity indexes (ALU115/ALU60 and ALU247/ALU60) have shown potential as non-invasive diagnostic biomarkers for OSCC. For instance, the median salivary DNA integrity, calculated as ALU115/ALU60, was higher in patients affected by OSCC [40]. These results indicate that analyzing cfDNA integrity, in addition to concentration, can provide valuable diagnostic and potentially prognostic information for OSCC and related head and neck cancers [40,47].

### 3.4. DNA Methylation Patterns and Epigenetic Signatures in cfDNA

Abnormal epigenetic modifications, particularly DNA methylation, are hallmarks of cancer and play a significant role in the development and progression of OSCC. Aberrant DNA methylation, especially hypermethylation of tumor suppressor genes (TSGs), is a well-documented event in OSCC tissues [49,50]. Detecting these methylation patterns in cfDNA from easily accessible body fluids like saliva and plasma offers a promising non-invasive approach for diagnosis and prognosis [51].

Several tumor suppressor genes have been found to be aberrantly hypermethylated in OSCC, including CDKN2A, p16, DAPK, RASSF1A, MGMT, E-cadherin, SFRP1, and TIMP3 [48,51,52,53]. Hypermethylation of these genes has been identified in OSCC tumor tissue and detected in corresponding cfDNA samples from saliva and plasma. For instance, promoter methylation of P16, DAPK, and RASSF1A has been found to be significantly hypermethylated in serum of OPSCC patients compared to normal controls, showing high concordance with tissue samples. Methylation of P16 was associated with smoking, while RASSF1A methylation correlated with stage [48,51]. TIMP3 hypermethylation in post-treatment salivary rinse samples has shown prognostic significance in HNSCC patients [53], and ZNF582 methylation in saliva has also been investigated as a potential biomarker for OSCC [54].

Beyond TSGs, the methylation status of genes involved in DNA methylation processes themselves, like DNMT3A and TET2, has been correlated with OSCC. Varying methylation degrees of DNMT3A and TET2 were revealed in OSCC tissues, and high DNMT3A methylation levels were associated with higher survival rates [49]. The detection and analysis of these specific DNA methylation patterns and broader epigenetic signatures in cfDNA hold significant potential as non-invasive biomarkers for early detection, diagnosis, prognosis, and perhaps even predicting treatment response in OSCC [50].

## 4. Clinical Applications and Translational Relevance

The emergence of liquid biopsy, particularly the analysis of cfDNA and ctDNA, represents a significant advancement in oncology, offering non-invasive approaches to cancer management [55]. This technology holds substantial translational relevance for oral carcinogenesis and OSCC, with potential applications spanning early detection, prognostic assessment, treatment monitoring, and guiding personalized therapy. Liquid biopsies can be performed on various bodily fluids, including blood plasma, saliva, and serum, providing a real-time window into tumor dynamics that traditional tissue biopsies cannot fully capture due to factors like intra- and inter-tumor heterogeneity [56].

### 4.1. CfDNA for Early Detection and Prognostic Stratification

Early detection remains critical for improving outcomes in HNSCC, which is often identified at advanced stages [57]. CfDNA has been explored in blood and saliva samples from HNSCC patients, and specific molecular alterations detectable in cfDNA, such as DNA methylation patterns, are being investigated as potential biomarkers for early disease identification [58,59]. For instance, methylation of tumor suppressor genes like *PAX5* has been assessed in oral rinses of HNSCC patients for early detection and monitoring, and *PAX5* has demonstrated encouraging specificity and sensitivity in oral rinses [59]. DNA methylation is considered an early event in cancer development and progression, and its analysis in cfDNA holds promise for diagnostics, although further highlights and investigations are needed [58,60]. Research also suggests that cfDNA coverage patterns at transcription start sites (TSSs) can reflect gene expression and have potential in early cancer screening [61].

Beyond early detection, cfDNA and ctDNA show promise for prognostic stratification in OSCC and HNSCC [57,62], and elevated cfDNA levels have been correlated with poor prognosis in various cancers, including OSCC [62]. Higher total cfDNA concentrations have been linked to worse overall survival in pancreatic ductal adenocarcinoma and prostate cancer, and the baseline concentration of cfDNA has been identified as an independent prognostic factor [63,64]. Moreover, increased plasma cfDNA has been suggested as a potential marker for oral cancer [42]. A study in OSCC patients has shown that a rapid increase in total cfDNA concentration can predict systemic metastases, and analysis of total cfDNA concentrations may be useful in predicting patient prognosis. The concentration of total cfDNA has also been reported to correlate directly with the extent of disease progression in several cancer types [65]. Furthermore, specific characteristics of cfDNA, such as fragmentation patterns and size distributions, are being investigated for their potential diagnostic and prognostic value [66].

### 4.2. Monitoring Therapeutic Response and Recurrence

Circulating tumor DNA analysis allows for real-time monitoring of tumor dynamics and treatment response, which is particularly valuable in HNSCC given the challenges in discriminating residual or recurrent disease post-treatment [56,67]. Longitudinal monitoring of ctDNA can assess minimal residual disease (MRD) after curative-intent therapy, and the presence of MRD detected by ctDNA assays after treatment has been shown to predict progression-free survival and overall survival in locally advanced HNSCC, often without the need for tumor sequencing [68]. Patients who remain negative for ctDNA after initial curative treatment tend to have a significantly better prognosis than those who test positive again [69]. Changes in ctDNA levels throughout treatment correlate with therapeutic efficacy and can precede clinical detection of recurrence or progression [62]. For instance, monitoring the methylation levels of specific markers in oral rinses has been suggested as an effective way to predict recurrence in HNSCC [59]. Similarly, rapid clearance profiles of plasma circulating tumor HPV DNA during chemoradiotherapy correlate with disease control in HPV-associated oropharyngeal cancer, and detectable postoperative circulating tumor HPV DNA is associated with recurrence [70]. CtDNA kinetics during treatment with immune checkpoint blockade (ICB) can potentially offer real-time assessment of disease burden [71]. Individualized ctDNA monitoring using specific gene panels and techniques like digital PCR (dPCR) is being explored for its clinical validity in monitoring treatment response and relapse in HNSCC [69]. Analyses of fluctuations in total cfDNA concentration can also help predict recurrence or metastasis in OSCC [65].

### 4.3. CfDNA as a Companion Diagnostic Tool in Personalized Oncology

Liquid biopsy facilitates the identification of tumor-specific genetic alterations, including mutations and epigenetic changes like methylation, which are crucial for guiding personalized treatment strategies [72,73,74]. Unlike traditional tissue biopsies, liquid biopsies can better capture the genetic heterogeneity of the tumor, including primary sites and metastases [57]. Detecting actionable mutations through ctDNA analysis offers the opportunity to select patients who may benefit from targeted therapies [75]. Comprehensive genomic profiling assays using cfDNA can identify a wide range of genetic alterations [76], while TP53 is a frequently mutated gene in HNSCC, including OSCC, and its mutations can be detected in ctDNA, in which panels for HNSCC also include other frequently altered genes [44,69,77]. The ability to identify specific genetic alterations in ctDNA without the need for repeated tissue biopsies is particularly advantageous in the context of treatment decisions and managing resistance mechanisms [75]. Although the clinical application of cfDNA and ctDNA in oral cancer is still developing and requires further research for standardization, their potential as companion diagnostic tools in personalized oncology is significant [62]. In this regard, even if integrating cfDNA analysis into clinical practice has challenges, it presents opportunities for enhancing precision medicine in cancer care [65].

## 5. Challenges, Limitations, and Future Directions

Despite the promising potential of cfDNA as a prognostic biomarker in oral carcinogenesis and OSCC, several challenges and limitations must be addressed to facilitate its clinical translation, shaping the future directions of research in this field. A significant hurdle lies in technical and pre-analytical variables. Saliva, while easily collected non-invasively [38], presents challenges in sample collection, handling, and processing, impacting the yield and quality of cfDNA and ctDNA [78]. The concentration of cfDNA in saliva is relatively low, and its stability during storage and transportation without proper preservatives is a concern, risking degradation and contamination from cellular genomic DNA. Although technologies like ddPCR have improved sensitivity for low analyte volumes, optimizing preservation methods remains crucial for maximizing detectable cfDNA [38].

Standardization and validation in oral oncology are also critical needs. Current studies utilize varied techniques for sample collection, extraction, and analysis of biomarkers, leading to variability in results and hindering comparison and widespread clinical adoption [78,79,80,81]. In particular, significant analytical variability remains a critical barrier to the clinical adoption of cfDNA analysis. Differences in pre-analytical handling (e.g., blood vs. saliva samples, processing time, and storage conditions), cfDNA isolation techniques, and quantification methods can lead to inconsistent results. Additionally, variability in detection thresholds and assay sensitivity across platforms further complicates clinical interpretation and reproducibility. Standardization of protocols across laboratories and development of validated reference materials will be crucial for ensuring consistency and comparability in cfDNA-based diagnostics. Furthermore, establishing standardized protocols for collection, processing, analysis, and reporting of liquid biopsy findings is essential [20].

The biological complexity of the oral tumor microenvironment (TME) poses another challenge. HNSCC is characterized by a complex and heterogeneous mutational landscape and a dysregulated TME [82,83], which includes various cellular and non-cellular components that interact with tumor cells [84]. Lymph node metastasis, a major determinant of poor prognosis in OSCC, is influenced by the stromal cues within the lymph node microenvironment [85].

Integrating cfDNA with other biomarkers offers a promising future direction to assess new therapeutic strategies’ effectiveness and improve diagnostic and prognostic accuracy [86]. Analyzing multiple components from liquid biopsies, such as ctDNA, for genetic mutations and copy number alterations [87], cell-free RNA, including microRNAs, which play a crucial regulatory role in oncogenesis [20], and proteins or metabolites can provide a more comprehensive molecular profile of the tumor [88,89], potentially identifying multi-marker signatures with higher sensitivity and specificity than single markers [20,90].

Finally, longitudinal studies and prospective clinical trials are indispensable for evaluating the clinical utility of cfDNA and other liquid biopsy biomarkers. Monitoring changes in biomarker levels over time can provide insights into disease progression, response to therapy, and early detection of recurrence, which is often missed by conventional imaging methods at early stages [89,91]. Prospective trials comparing liquid biopsy approaches to existing standard methods, such as tissue biopsy and imaging, are needed to generate the evidence required for widespread clinical implementation and to validate their impact on patient outcomes [92,93]. Furthermore, the development of integrated platforms that combine cfDNA with other analytes, such as exosomal RNA or proteomic panels, could overcome current limitations in sensitivity and specificity. By capturing complementary molecular signals across different biological layers, these multi-analyte approaches may enhance early detection, improve disease stratification, and offer a more robust and comprehensive tool for personalized management of OSCC.

### Techniques and Challenges for cfDNA-Containing Sample Handling in Liquid Biopsy

The successful integration of cfDNA into clinical practice for liquid biopsy, particularly for HNSCC and OSCC, hinges on standardized protocols for sample collection, storage, and transport.

Regarding sample collection and preservation, blood samples are commonly used for liquid biopsies in solid malignancies and are typically collected in K2EDTA or specialized Streck Cell-Free DNA Blood Collection Tubes (cfDNA BCTs). Streck cfDNA BCTs can maintain cfDNA stability for at least 3 days, at 18–22 °C, making them suitable for transport. K2EDTA tubes, while common, generally require processing within 6 h at room temperature to prevent contamination from lysed blood cells [38]. Saliva is also a promising, non-invasive alternative, due to its association with cancer progression [94]. Collection methods include passive spitting into sterile tubes or using devices like Salivette or Oragene OG-600 receptacle, which can yield high concentrations of total salivary DNA [80,94].

cfDNA stability in saliva is a significant challenge, as unpreserved salivary cfDNA can degrade rapidly, losing over half within 24 h, with reported half-lives around 13–14 h at room temperature. In contrast, cfDNA in blood typically has a shorter half-life of 15 min to 2.5 h. Research indicates that UAS preservative can stabilize salivary cfDNA at room temperature for up to one week. Additionally, 20 mM EDTA can stabilize salivary supernatant cfDNA for up to one week if processed immediately after collection [38]. Salivary miRNAs have shown stability for up to 48 h at 4 °C in saliva supernatant, though significant alteration occurs after 96 h. Mid-term storage at −20 °C can decrease miRNA stability compared to the standard −80 °C, suggesting immediate processing and long-term storage at −80 °C is generally recommended for optimal preservation [78].

Beyond technical limitations, real-world barriers hinder the clinical implementation of cfDNA and ctDNA analysis in OSCC. High assay costs, the need for specialized equipment and trained personnel, and limited availability of next-generation sequencing platforms pose challenges, especially in low- and middle-income countries. Furthermore, regulatory frameworks for the approval and standardization of liquid biopsy tests in oral oncology remain underdeveloped. Addressing these practical and systemic hurdles will be essential for translating molecular diagnostics into widespread clinical practice.

## 6. The Emerging Role of Proteomics and Metabolomics in Oral Cancer Biomarker Discovery

**Figure 3:**
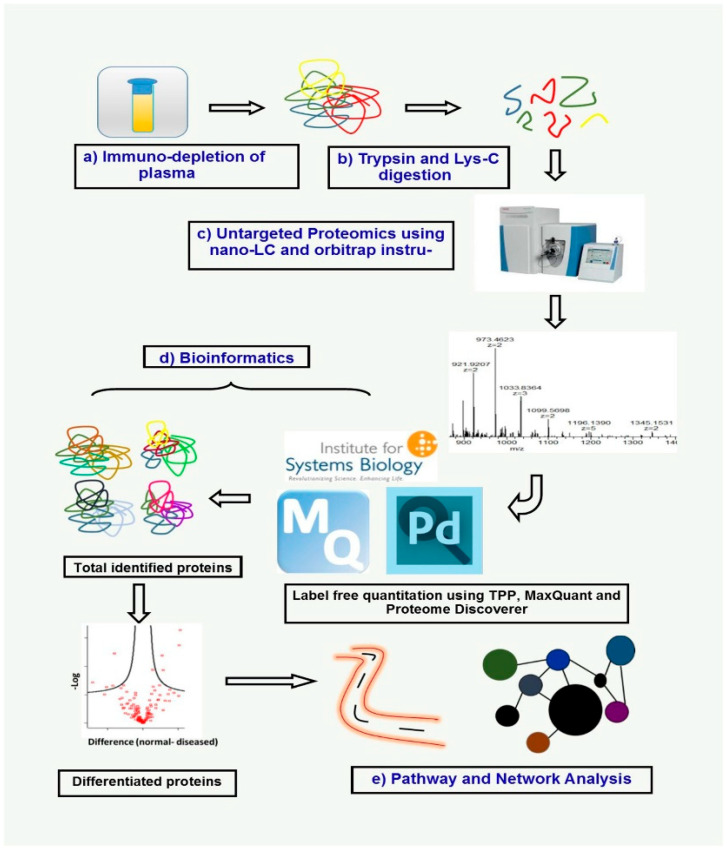
Plasma immunodepletion and label-free MS quantitation workflow: (**a**) Immunodepletion: Remove the top 12 abundant plasma proteins. (**b**) Proteolysis: Digest the depleted sample with Trypsin and Lys-C; then, wash and elute peptides. (**c**) Untargeted LC-MS/MS: Analyze peptides via nano-LC coupled to tandem MS to identify all detectable sequences. (**d**) Label-Free Quantitation: Process raw spectra in MaxQuant, Proteome Discoverer, and the Trans-Proteomic Pipeline; filter and statistically pinpoint differentially abundant proteins. (**e**) Functional Analysis: Subject those proteins to pathway and network analysis to elucidate their roles in disease biology. From [96] with permission.

OSCC is a prevalent malignancy of the head and neck region, often diagnosed at advanced stages, leading to an unfavorable prognosis. The absence of precise and efficient early diagnostic techniques remains a key challenge in OSCC management. In recent years, “omics” technologies, such as genomics, proteomics, and metabolomics, have been leveraged to identify clinically relevant biomarkers in various biological fluids and tissues of OSCC patients. These approaches offer promising non-invasive strategies for early detection, prognostication, and monitoring of the disease (Figure 3) [95,96].

### 6.1. Proteomics in Oral Cancer Biomarker Discovery

Proteomics involves the large-scale study of proteins, providing insights into cellular functions and physiological states. Mass spectrometry (MS)-based proteomics has become a powerful analytical tool for identifying molecular markers. Among the various biofluids, saliva is extensively used as a preferred biofluid for identifying novel biomarkers for OSCC. This preference is due to its non-invasive collection method, its proximity to oral cancer lesions, and its relative simplicity compared to tissue biopsies or blood [96].

Several studies have applied MS-based proteomics (e.g., iTRAQ, LC-MRM/MS, MALDI-MSI, and SELDI-TOF/MS) to profile saliva from OSCC, OPMD, and healthy subjects [95,96,97,98,99]. For instance, a study found 67 proteins up- and 18 down-regulated in OSCC versus non-cancerous samples; three candidates (CFH, FGA, and SERPINA1) were confirmed by targeted MRM-MS and immunoassays and correlated with advanced disease stage [99].

Beyond saliva, proteomic analysis has also been applied to other samples related to oral cancer, such as serum, plasma, tumor tissue, and the TME [100,101]. A systematic review of proteomic studies in the OSCC TME identified 570 proteins detected by MS-based proteomics, with 28 being cited by two or more studies. These frequently identified proteins participate in extracellular matrix remodeling and/or energy metabolism, and most share the feature of being modulated by hypoxia in the proliferating OSCC mass [101].

In this regard, the analysis of proteomics in saliva and blood, often focusing on exosomal proteins, is considered helpful in the assistance of diagnosis and personalized treatments of OSCC [102] and, overall, analysis of salivary proteome and proteomic studies of the TME demonstrate feasibility and significant potential for discovering biomarkers for oral cancer [99,101].

### 6.2. Metabolomics in Oral Cancer Biomarker Discovery

**Figure 4:**
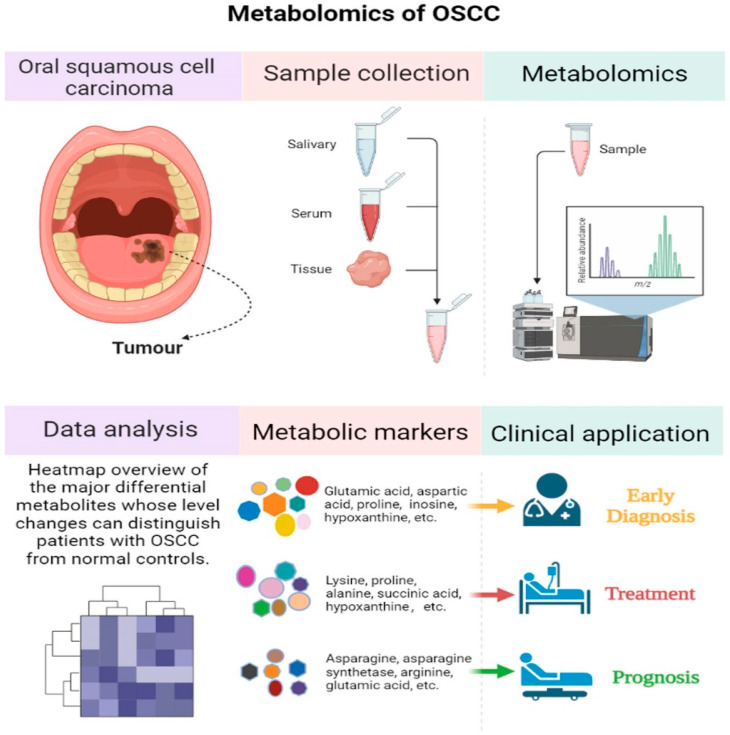
The application of metabolomics in oral squamous cell carcinoma research. This figure was created using biorender.com. From [95] with permission.

Metabolomics involves the comprehensive study of small molecule metabolites within biological systems [103]. Similar to proteomics, salivary metabolomics has emerged as a promising non-invasive approach for discovering biomarkers for oral cancer and OPMDs [102]. Techniques such as gas chromatography–mass spectrometry (GC-MS), ultra-high-performance liquid chromatography–tandem mass spectrometry (UHPLC-MS/MS), capillary electrophoresis–mass spectrometry (CE-MS), and nuclear magnetic resonance (NMR) spectroscopy are commonly employed to identify and quantify salivary metabolites [95,104,105]. Metabolomics detection technology can efficiently identify differential metabolites (Figure 4) [95]. Studies have shown significant alterations in salivary metabolites in OSCC patients compared to healthy individuals and, importantly, differences can also be observed between OSCC and OPMD patients and even between OSCC and other oral conditions like oral lichen planus [106,107,108].

These metabolic changes often reflect impaired metabolic pathways associated with cancer development and progression, such as choline metabolism, amino acid pathways, polyamine metabolism, the urea cycle, creatine metabolism, glycolysis, and glycerolipid metabolism [106]. Specific metabolites frequently reported in salivary metabolomics studies of oral diseases include glycine, leucine, phenylalanine, dipeptides, linoleic acid, arachidonic acid, tyrosine, choline, taurine, lactate, valine, and proline [103]. Studies on OSCC tissue metabolomics have also provided insights, revealing changes like decreased levels of certain amino acids (glycine, serine, glutamine, lysine, proline, alanine, glutamic acid, leucine, and threonine) from healthy tissue to precancerous lesions and then to OSCC. The enhanced consumption of glucose and lactate production (the Warburg effect) has also been observed in OSCC tissues through metabolomic analysis [95]. Salivary metabolomics, especially when combining analyses of different metabolites, shows feasibility for the diagnosis and monitoring of OSCC [102].

While some salivary metabolites exhibit significant changes in OPMDs and oral cancer compared to healthy controls, further high-quality studies are needed to confirm their reliability and establish their diagnostic potential [106]. Integrating metabolomics with statistical data analyses and artificial intelligence technology holds promise for rapid screening of characteristic biomarkers for early diagnosis, treatment, and prognosis evaluations (Table 1) [95].

## 7. Conclusions

This review highlights the growing relevance of liquid biopsy approaches, particularly cfDNA and ctDNA analysis, in the early detection, prognosis, monitoring, and personalized treatment of OSCC. CfDNA, especially when analyzed for specific methylation patterns or concentration changes, shows potential as a non-invasive biomarker for early diagnosis and risk stratification. CtDNA offers real-time insights into tumor dynamics, enabling the detection of minimal residual disease and recurrence monitoring. The integration of these biomarkers with digital PCR and gene panels enhances their clinical utility in tracking disease burden and guiding therapy decisions. Moreover, the expanding roles of proteomics and metabolomics, particularly in saliva, have led to the identification of promising protein and metabolite signatures that may improve diagnostic precision and prognostic accuracy in OSCC and OPMDs.

The application of cfDNA and ctDNA in OSCC represents a shift towards precision oncology, with the potential to complement or even replace traditional invasive diagnostic tools. However, challenges remain in standardizing sample processing, ensuring analyte stability, especially in saliva, and addressing variability in analytic platforms. Further, the biological complexity of the TME necessitates a multimodal biomarker approach. Combining cfDNA with other biomolecules such as microRNAs, exosomal proteins, and metabolites may provide a more comprehensive tumor profile. The utility of proteomic and metabolomic analyses in salivary samples reinforces the feasibility of non-invasive diagnostics and offers valuable insights into tumor biology and progression. Nonetheless, high-quality longitudinal studies and prospective clinical trials are essential to validate these biomarkers and translate them into standardized clinical workflows. Future research developing integrated and multiplexed platforms is needed to develop reliable and accurate interpretation and early intervention in oral carcinogenesis.

## Figures and Tables

**Table 1:** Metabolic markers for early diagnosis of OSCC. From [95] with permission.

Sample	Comparison	Metabolite Analysis Technique	Differential Metabolites	References
Saliva	OSCC versus OLK	GC-TOF-MS	Tyrosine, malic acid, tyrosine, lactic acid, 2-hydroxybutyric acid, prostaglandin E2	Xue et al. [109]
	OSCC versus HC	GC-TOF-MS	Prostaglandin E2, lactic acid, tyrosine, lactic acid, 2-hydroxybutyric acid, prostaglandin E2	Ferrarini et al. [110]
	OLK versus HC	GC-TOF-MS	Tyrosine, lactic acid, 2-hydroxybutanedioic acid, prostaglandin E2	Xue et al. [109]
	OSCC versus OLK	GC-MS	Decanedioic acid, 2-methyloctadecane, eicosanoic acid, octane, 3,5-dimethyl	Tantray et al. [111]
	OSCC versus HC	CPSI-MS	Polyamines (e.g., spermidine, spermine) and amino acids (e.g., arginine, lysine, histidine, glutamine, leucine) Metabolites related to energy metabolism (e.g., glucose, creatine, creatinine) and purine metabolites (e.g., inosine, hypoxanthine)	Song et al. [112]
	HC versus PML	CPSI-MS	Inosine, hypoxanthine, adenosine, thymidine, uridine, guanosine, cytosine, choline, sphingolipids, etc.	Song et al. [112]
Serum	OSCC versus HC	UHPLC-Q-Orbitrap HRMS	LysoPC, taurine, and glutamate	Li et al. [113]
	OSCC versus HC	CPSI-MS	GPC, lyso-GPC, acylcarnitine, DG, sphingolipids, etc.	Yang, Song, Yang, et al. [114]
	OSCC preoperative versus postoperative	UHPLC-Q-Orbitrap HRMS	Succinic acid, arginine, L-carnitine, NAcetyl-L-tyrosine, glutamine, xanthine, sphingosine, palmitoyl ethanolamide, hexanoyl carnitine, whey acid, uric acid, vanillylmandelic acid, ethyl acetate, thromboxane B2	Zuo et al. [115]
Tissue	OSCC versus HC	GC–MS UHPLC–MS/MS	Glutamic acid, aspartic acid, proline	Yang et al. [116]
	OSCC versus PML versus HC	GC-MS Analyses	Glycine, threonine, glutamine, lysine, proline, alanine, glutamic acid, leucine, serine	Musharraf et al. [117]
	OSCC versus HC	CE-TOF-MS	Glucose, glycerol triphosphate, glycerol diphosphate, lactic acid	Ogawa et al. [118]

## Data Availability

Not applicable.

## Abbreviations

OPMDs	Oral potentially malignant disorders
OSCC	Oral squamous cell carcinoma
Cf-DNA	Cell-free DNA
Ct-DNA	Circulating tumor DNA
OLK	Oral leukoplakia
OLP	Oral lichen planus
OSF	Oral submucous fibrosis
CSF	Cerebrospinal fluid
HNSCC	Head and Neck Squamous Cell Carcinoma
ddPCR	Droplet digital polymerase chain reaction
WES	Whole-exome sequencing
MRD	Minimal residual disease
dPCR	Digital PCR
TME	Tumor microenvironment
MS	Mass spectrometry
iTRAQ	Isobaric tags for relative and absolute quantitation
LC-MRM/MS	Liquid Chromatography Multiple-Reaction-Monitoring Mass Spectrometry
MALDI-MSI	Matrix-assisted laser desorption/ionization mass spectrometry imaging
SELDI-TOF/MS	Surface-enhanced laser desorption/ionization-time-of-flight/mass spectrometry
CFH	Complement factor H
FGA	Fibrinogen alpha chain
SERPINA1	Alpha-1-antitrypsin
GC-MS	Gas chromatography–mass spectrometry
UHPLC-MS/MS	Ultra-high-performance liquid chromatography–tandem mass spectrometry
CE-MS	Capillary electrophoresis-mass spectrometry
NMR	Nuclear magnetic resonance
